# Investigating Patient Perspectives on Using eHealth Technologies for the Self-Management of Inflammatory Bowel Disease: Mixed Methods Study

**DOI:** 10.2196/53512

**Published:** 2024-09-06

**Authors:** Sander Hermsen, Danielle Tump, Eva Wentink, Marjolijn Duijvestein

**Affiliations:** 1 OnePlanet Research Centre Precision Health and Nutrition Group Wageningen Netherlands; 2 Radboud University Medical Centre Prevention Hub Nijmegen Netherlands; 3 Radboud University Medical Centre Department of Gastroenterology Nijmegen Netherlands

**Keywords:** information and communication technology, ICT, self-management, inflammatory bowel disease, IBD, smart toilet seat, mobile phone

## Abstract

**Background:**

Inflammatory bowel disease (IBD) poses significant challenges for patients, requiring continuous monitoring and self-management to improve quality of life.

**Objective:**

This study aims to investigate the viewpoints of individuals living with IBD on the use of information and communication technology (ICT) for the self-management of their condition, with a particular focus on the concept of a “smart” toilet seat as an example of ICT for IBD self-management.

**Methods:**

We conducted an analysis of questionnaire responses obtained from 724 participants. They were encouraged to share their use cases and identify any perceived barriers associated with ICT adoption for managing their condition. To assess their responses, we used descriptive quantitative analysis, summative content analysis, and thematic qualitative analysis. We combined these results in an epistemic network analysis to look for meaningful patterns in the responses.

**Results:**

Of the 724 participants, more than half (n=405, 55.9%) were already using various forms of ICT for IBD self-management. The primary factor influencing their use of ICT was their affinity for interacting with technology. Distinct differences emerged between individuals who were using ICT and those who were not, particularly regarding their perceived use cases and concerns.

**Conclusions:**

This study provides valuable insights into the perspectives of individuals with IBD on the use of ICT for self-management. To facilitate wider adoption, addressing privacy concerns, ensuring data security, and establishing reliable ICT integration will be critical.

## Introduction

### Background

Inflammatory bowel disease (IBD), an umbrella term for chronic gastrointestinal diseases, including Crohn disease (CD) and ulcerative colitis (UC), affects approximately 0.3% of the population in North America and Oceania, 0.2% of the population in Europe, and rapidly rising proportions of the population in many other countries around the world [1,2]. The rising incidence has resulted in increased health care use and longer outpatient waiting lists [3]. One way to alleviate pressure on the health care system is to reduce waiting lists and health care use through patient self-management, that is, a patient’s “ability to manage the symptoms, treatment, physical and psychosocial consequences, and lifestyle changes inherent in living with a chronic condition” [4]. Being a chronic condition, IBD entails lifelong self-management; even in remission, IBD is known to have a severe impact on mental health, with recent evidence suggesting that approximately 30% of people with IBD report symptoms of anxiety or depression [5-7]. Self-management, including the patient’s ability to monitor their condition, is not only effective in relieving health care but is also well known to effectively maintain or even increase quality of life [4,8]. Self-management strategies are usually related to diet and the avoidance of perceived trigger foods [9-11], seeking support, stress reduction, experimentation, and lifestyle adaption [11,12].

In recent decades, information and communication technology (ICT) has played an ever-increasing role in the self-management of chronic conditions [13,14]. People living with IBD have been found to be generally willing to use ICT solutions for the self-management of their condition [15]. ICT solutions used for IBD self-management include websites [16]; smartphone apps [17,18]; wearable sensors [15,19]; and, lately, domotics such as “smart” toilet seats [20,21], which may soon arrive in the consumer market. Compared with standard outpatient-led care, ICT solutions (also known as eHealth technologies) have been shown to lead to improvements in relapse duration, disease activity, short-term medication adherence, quality of life and stress reduction, IBD knowledge, health care costs, and clinic visits [22].

Unfortunately, the efficacy of a majority of current ICT solutions for IBD self-management is marred by a lack of professional medical involvement and the limited use of evidence and international consensus guidelines [23]. Another important detrimental factor is a lack of patient input to inform the development of ICT solutions. Patient input is important because research shows that patients with IBD desire more information and actionable strategies with regard to managing their disease, shared decision-making, and reducing the burden of treatment and symptom control [24,25]. Furthermore, although using ICT for IBD self-management improves participants’ feelings of empowerment [25], the views, needs, and desires of users, as well as the barriers they face, have as yet largely been disregarded [15,21,26]. One recent study sheds light on patient self-management strategies and needs for ICT intervention content [27], but much remains unclear about prerequisites, desires, and barriers to using ICT for the self-management of IBD, such as individual differences in, and determinants for, the use of ICT; the impact of self-management strategies and needs on the use of ICT; current ICT use; and perceived use cases.

### Objectives

This study addresses the lack of patient perspectives on using ICT for the self-management of IBD in the literature by researching self-management strategies and needs, current ICT use, perceived ICT use cases, and the perceived efficacy of patients with IBD, and individual differences in ICT use in self-management. The study further elucidates perceived use cases, concerns, and barriers for a novel method to support IBD self-management by presenting participants with a description of a smart toilet seat currently under development at OnePlanet Research Center in Wageningen, Netherlands. Using low-fidelity prototypes such as descriptions or paper examples enables people to reflect on their potential use cases and barriers for ICT for IBD self-management [28,29]. The literature [21,30] shows that people differ in their use cases for smart technology, ranging from documenting aspects of their condition and using technology as a tool for diagnosis to signaling exacerbations and prescribing a course of action. Furthermore, people differ in terms of concerns and barriers, such as the transparency and visibility of technology use [31], privacy, and data agency (ie, who controls the dissemination of the data) [21]. What determines these differences, how they are interrelated, and how they relate to self-management strategies as yet remain unknown. This study contributes to addressing this gap in our knowledge.

## Methods

### Overview

To investigate the perspectives of people with IBD on using technology for the self-management of their condition and to identify the use cases and barriers for people with IBD regarding a smart toilet seat for IBD self-management, we performed a mixed methods study. We used a 3-part web-based questionnaire with both Likert-scale questions and questions with open-text fields. The first part of the questionnaire contained questions on demographics, health status, and disease self-management; the second part covered the use of ICT for disease self-management; and the third part focused on potential use cases and barriers for a specific ICT example, that is, a smart toilet seat. Responses to the Likert-scale questions were analyzed using descriptive statistics, correlations, and binomial general linear modeling, and these served as criteria for stratification in the qualitative analyses of the open-text questions. The answers to the open-text questions were analyzed and coded using summative content analysis and thematic qualitative analysis [32,33]. We then combined the quantitative and qualitative analyses through epistemic network analysis (ENA) [34-37], which enabled us to evaluate differences in the responses as well as their patterns across the stratification groups.

### Participants

The study participants were members of the patients’ association Crohn & Colitis NL (CCNL [38]). CCNL has >10,000 members and sends out a monthly email newsletter. In this newsletter, we published a brief invitation to participate in the questionnaire, which was sent out to 2274 members, of whom 1633 (71.81%) identified as female, 634 (27.88%) as male, and 6 (0.26%) as other or unknown. The average age of the participants was 48.77 (SD 15.72) years. Of the 2273 members, 568 (24.98%) had UC, 707 (31.09%) had CD, and 999 (43.93%) had not disclosed their condition when subscribing to the CCNL newsletter. The inclusion criteria for participation were as follows: having IBD (CD, UC, or as-yet-undiagnosed IBD), being aged ≥18 years, and having access to the internet and email. There were no further inclusion or exclusion criteria.

Sample size considerations were driven by the qualitative analyses. A priori sample size calculations in qualitative research are subject to conceptual debate and practical uncertainty [39]. Saturation (ie, the moment when adding more data does not lead to new insights) is often seen as a criterion for whether to include more participants once analysis has started. Although saturation as a concept is contested [39], it is still the most practical measure of information maturity. As a rule of thumb, 20 to 40 participants are usually considered sufficient to achieve saturation [40]. Another measure is information power [41]: the density, depth, and specificity of the information required affects the required sample size. In this study, the aim was relatively broad, the combination of participants was less specific for the research question, there was little theory to inform the analysis, the questionnaire methodology allowed for little dialogue, and we planned to perform cross-case analyses. Therefore, we could expect the need to include more participants for a mature data set than rule-of-thumb approaches would suggest. For the ENA approach, no sample size considerations exist as yet, but a rule-of-thumb consideration is that there should be more participants than codes in each network [33-37], and it is advisable to achieve thematic saturation within each network. In this study, the maximum number of potential networks to compare would be when comparing age groups (5 groups, resulting in 5 networks). Therefore, we aimed to include at least 200 participants—5 times as many as rules of thumb would advise—to make ENA comparisons between groups possible.

### Procedure

After clicking the link in the newsletter, participants were taken to the questionnaire platform. Before entering the questionnaire, participants were presented with a consent form and asked to provide informed consent. They could exit the questionnaire at any time for any reason without any consequences. Participant data were only stored when they clicked the Send Results button at the end of the questionnaire. However, participants had the option to skip questions; any unanswered questions were recorded as missing data. Filling out the questionnaire took approximately 20 minutes. The questionnaire remained accessible for 3 weeks after participants received the invitation.

### Ethical Considerations

This study was deemed exempt from ethics approval under Dutch law by the ethics review board of the Máxima Medical Centre, Veldhoven (N23.030). Participants provided informed consent before starting the questionnaire by reading and agreeing to a statement outlining the goal and contents of the questionnaire. They were informed that all data would be stored anonymously, that answers would not be analyzed on an individual level, and that they had the right to skip any question. The statement also explained that filling out the questionnaire meant providing consent for the use of their questionnaire data for analysis and in scientific and professional publications. Finally, the statement advised them that they could quit participating at any time by closing the questionnaire and refraining from submitting their responses; no data were stored before the questionnaire was completed. Neither CCNL nor the researchers had access to any names or physical or email addresses of participants. After questionnaire completion, data that could potentially pose a privacy risk, such as age and occupation details, were pseudonymized by converting quantitative data into categorical data. No compensation was offered to participants for taking part in the study.

### Reflexivity

In research aimed at understanding participants’ experiences, there is a potential risk of researcher bias [42]. To enhance the integrity and credibility of qualitative research, researchers must assess how intersubjective components affect data collection and analysis. An instrument for this examination is reflexivity, which refers to researchers’ explicit, self-aware appraisal of their own roles [42,43].

The host institute of this study, OnePlanet Research Center, is a relatively new (it was founded 5 years ago) research institute focused on potential innovations in health and sustainability using sensor technology and artificial intelligence. One of its research programs is dedicated to gut health, with the smart toilet seat described in this study being an important part. The end goal of the program is the development of an integrated suite of sensors that informs a personal digital twin model designed to signal, measure, and prevent a range of health conditions.

SH is the principal behavioral scientist at OnePlanet Research Center, where he leads human factors research. His work focuses on the acceptability, usability, and efficacy of technological innovations designed to support healthy living. DT is a senior biomedical researcher at OnePlanet Research Center and is responsible for research projects within the smart toilet seat project. EW is the principal investigator of the smart bathroom program at OnePlanet Research Center; she leads all scientific and developmental activities for the smart toilet seat and other innovations. MD is the head of clinical research at the department of gastroenterology, Radboud University Medical Centre, Nijmegen. She specializes in treating IBD.

### Questionnaire

Participants filled out a 3-part questionnaire containing 31 questions on demographics; health status and disease self-management; the use of ICT for disease self-management; and potential use cases and barriers for a specific ICT example, that is, a smart toilet seat (refer to Textbox 1). The full questionnaire, translated into English, is available in Multimedia Appendix 1.

**Brief details of the questionnaire. Open-text questions for qualitative analysis are displayed in italics.**

**Questionnaire part 1 (questions 1-17)**

- Demographic data: age, sex, education level, and occupation category
- Health status: condition type (Crohn disease, ulcerative colitis, or as-yet-undiagnosed inflammatory bowel disease), years since diagnosis, the effect of the condition on everyday life, *perceived symptoms, comorbidities*, and recent exacerbations (the questions on symptoms and comorbidities were presented as long lists, where participants could mark checkboxes for their symptoms or comorbidities and then supplement the list with their own experiences in an open-text field)
- Disease self-management: ability to predict an exacerbation (flare), *predictors of exacerbations*, knowing the potential cause of the exacerbation, *perceived causes of exacerbations*, perceived influence on exacerbations, *self-management strategies*, and *self-management needs* (these questions were asked only if the participant indicated having had an exacerbation in the past year)

**Questionnaire part 2 (questions 18-23)**

- The use of information and communication technology (ICT) for condition self-management: short version of the Affinity for Technology Interaction questionnaire [44], the use of ICT for self-management, *the type of ICT used*, *perceived support for self-management through ICT, reasons not to use ICT, and perceived uses of ICT for self-management* (these questions were asked only if the participant indicated having had an exacerbation in the past year)
- A short description of a smart toilet seat, currently in development at OnePlanet Research Center; the description, which contained images of a toilet seat equipped with sensors, read (in Dutch): “Currently, OnePlanet Research Center is developing a special toilet with which you can, in the future, measure and observe all kinds of processes. The toilet performs its measurements automatically, all you need to do is sit down as on a regular toilet. In the toilet seat and elsewhere are small instruments that can measure, for instance, your heartbeat, breath, body temperature or urine or stool composition. By simply using the toilet, you can find out many things about your health. You can use an app or your smartphone, or the toilet can make a direct connection with your doctor”

**Questionnaire part 3 (questions 24-31)**

- Questions about the smart toilet seat: desire to use, perceived efficacy, *perceived use cases for the smart toilet seat, perceived barriers, data agency, who can receive data*, and *concerns*; finally, participants had the opportunity to leave a comment about the questionnaire

### Analysis

Data analysis consisted of 3 steps: quantitative analysis of the results of the questions used for stratification, summative content analysis and thematic qualitative analysis of the open-text question results, and ENA of the qualitative data using the quantitative results as stratification factors.

### Quantitative Analyses

Quantitative analyses were performed using RStudio for Mac (version 2023.03.0+386; Posit Software, PBC) [45]. The questionnaire data were first imported into RStudio. Where necessary, data were converted into factorial format and labeled. For numerical data, we calculated means, SDs, minimum and maximum values, and medians. For all data, we printed bar plot histograms for visual information. We then calculated the heterogenous correlation matrix, consisting of Pearson product-moment correlations between numeric variables, polyserial correlations between numeric and ordinal variables, and polychoric correlations between ordinal variables. Next, we qualified the correlations by testing binomial general linear models for the 2 main research questions: what predicts the use of ICT for self-management? What predicts willingness to use the smart toilet seat innovation? Finally, all quantitative data were gathered in a data frame for use as stratification factors in the ENA step, using the *source_from_df* function from the *rock* package for R [46].

### Qualitative Analyses

All source files were cleaned using the *clean_sources* function from the *rock* package. For each participant, answers to open-text questions were then grouped together per questionnaire part: (1) condition characteristics (ie, symptoms and comorbidities) and self-management (ie, predictors of exacerbations, perceived causes, self-management strategies, and self-management needs), (2) ICT use for IBD self-management (ie, which hardware and software participants currently use, perceived support for self-management through ICT, reasons not to use ICT, and desired uses of ICT), and (3) the smart toilet seat (ie, perceived use cases of the toilet, perceived barriers, data agency, who can receive data, and concerns).

All data were then manually segmented into utterances, and given a unique utterance identifier through the *prepend_ids_to_sources* function from the *rock* package. One researcher performed a summative content analysis [47] on the questions on symptoms, comorbidities, predictors of exacerbations, perceived causes, self-management strategies, self-management needs, and current technology use. Summative content analysis is well suited for counting and comparing keywords within content [47]. The researcher then performed a thematic analysis [32,33] on the results from the remaining open-text questions—*perceived support for self-management through ICT*, *reasons not to use ICT*, and *desired uses of ICT for self-management* from questionnaire part 2; *perceived use cases*, *perceived barriers*, *data agency*, *who can receive data*, and *concerns* from questionnaire part 3; and the open-text comment section—by coding and recoding all utterances using the i.rock web-based coding tool, a tool for creating human- and machine-readable coded qualitative data and then identifying themes within the codes. Thematic analysis is well suited to identify and interpret patterns of meaning within open qualitative data [33]. All coded utterances were then gathered in a data frame to use as analysis units in the ENA step, using the *parse_sources* function from the *rock* package.

### Combined Analysis: ENA

To look for meaningful patterns and differentiations between subgroups in the qualitative data, we performed an ENA using the ENA web tool (version 1.7.0) [48,49]. ENA is a technique for modeling the structure of connections in qualitative data. It assumes that (1) it is possible to systematically identify a set of meaningful features in the data (*codes*), (2) the data have local structure (*conversations*), and (3) an important feature of the data is the way that codes are *connected* to one another within conversations [34-37]. ENA models the connections among codes by quantifying the co-occurrence of codes within a given moving stanza window in the conversations (in our analysis, this moving stanza window was set to 6 utterances), producing a weighted network graph in which nodes correspond to the codes, and edges reflect the relative frequency of co-occurrence (or connection) between 2 codes. The positions of the network graph nodes are fixed, and those positions are determined by an optimization routine that minimizes the difference between the plotted points and their corresponding network centroids. Because of this coregistration of network graphs and projected space, the positions of the network graph nodes—and the connections they define—can be used to interpret the dimensions of the projected space and explain the positions of plotted points in the space. Furthermore, the size of the nodes indicates the importance of the code for the network. We created graphs by selecting all utterances from each unique participant as *conversations*; the codes associated with one of the themes derived from the thematic qualitative analysis as *codes*; and the different values of the stratification factors derived from the quantitative analysis, plus the unique participants, as *units*.

## Results

### Demographics, Health Condition, and Self-Management

Of the 2274 potential respondents, 724 (31.84%) participants with IBD filled out the questionnaire (ie, approximately 0.8%-1% of all people with IBD in the Netherlands) [50]. Participant demographic information (sex, age, and education; questions 1-3) is presented in Table 1. The results of the questionnaire are discussed in the subsections that follow; the results of part 1 (condition characteristics and self-management) are available in Table 1 (participant characteristics; questions 1-3), Table 2 (condition characteristics; questions 5, 6, and 10), Table 3 (differences in symptoms between participants with CD and those with UC; questions 8 and 9), and Table 4 (self-management skills, needs, and strategies; questions 11-17). The results of part 2 (ICT use for IBD self-management) are available in Table 5 (hardware and software use; questions 18-20). The results from the thematic analyses and ENAs on perceived use cases for ICT (questions 21-23) and the smart toilet seat (questions 24-31) are presented in detail in the following subsections. As the ENA may be unfamiliar to many readers, and it results in a large number of outcomes that may be difficult to interpret, we have added bullet points summarizing the main findings in each ENA subsection. An overview of all main themes, subthemes, codes, and code frequencies is available in Multimedia Appendix 2.

**Table 1:** Participant characteristics (questions 1-3; n=724).

Characteristics		Participants, n (%)
**Sex**		
	Female	489 (67.5)
	Male	233 (32.2)
	Other	0 (0)
	Not answered	2 (0.3)
**Age group (y)**		
	<31	76 (10.5)
	31-40	109 (15.1)
	41-50	117 (16.2)
	51-65	261 (36)
	>65	161 (22.2)
**Highest education achieved**		
	Primary school	5 (0.7)
	Primary vocational education	219 (30.2)
	Secondary vocational education	72 (9.9)
	Higher professional or university education	423 (58.5)
	Not answered	5 (0.7)

**Table 2:** Condition characteristics (questions 5, 6, and 10; n=724).

Characteristics				Participants, n (%)
**Condition type**				
	**Ulcerative colitis**		304 (42)	
		Female	182 (59.9)	
		Male	122 (40.1)	
	**Crohn disease**		394 (54.6)	
		Female	292 (74.1)	
		Male	102 (25.9)	
	**As-yet-undiagnosed inflammatory bowel disease**		24 (3.3)	
		Female	15 (62.5)	
		Male	9 (37.5)	
		Not answered	2 (0.03)	
**Years since diagnosis**				
	<1		36 (5)	
	1-5		128 (17.7)	
	6-10		134 (18.5)	
	10-25		237 (32.7)	
	>25		185 (25.6)	
	Not answered		4 (0.6)	
**Exacerbations in past year**				
	I have had an exacerbation		389 (53.7)	
	I have not had an exacerbation		283 (39.1)	
	I do not know		52 (9.9)	

**Table 3:** Symptoms for which participants with ulcerative colitis (UC) and those with Crohn disease (CD) mentioned different occurrences (questions 8 and 9; n=724).

Participants’ condition	Blood and slime in stool, n (%)	Tenesmus, n (%)	Frequent toilet visits, n (%)	Pain in joints, n (%)	Stomachache, n (%)
UC (n=304)	220 (72.4)	116 (38.4)	228 (75)	94 (30.9)	186 (61.2)
CD (n=395)	170 (43)	79 (20)	238 (60.2)	156 (39.5)	280 (70.9)

**Table 4:** Self-management skills (questions 11-17; n=283, ie, all participants who indicated having had an exacerbation in the past year).

Self-management skills			Participants, n (%)
**Perceived ability to detect exacerbations**			
	Never	86 (30.6)	
	Sometimes	135 (48)	
	Often	47 (16.7)	
	Always	13 (0.1)	
	Not answered	2 (0.1)	
**Knowing what causes exacerbations**			
	No	79 (27.9)	
	Yes	203 (71.7)	
**Can influence an exacerbation**			
	No	184 (65)	
	Yes	99 (35)	
**Exacerbation predictors**			
	Stomachache	71 (25.1)	
	Fatigue	70 (24.7)	
	Change in stool or diarrhea	41 (14.4)	
	Increased toilet visits	37 (13.1)	
	Stress	32 (11.3)	
**Perceived exacerbation causes**			
	Stress	158 (55.8)	
	Diet and trigger foods	105 (37.1)	
	Work and life pressures	45 (15.9)	
**Self-management needs**			
	Social support	105 (37.1)	
	Informational support	81 (28.6)	
	Resilience and optimism	64 (22.6)	
	Action planning or practical	35 (12)	
	No need for support	35 (12)	
**Self-management strategies**			
	Sleep and relaxation	65 (22.9)	
	Healthy diet, avoid triggers	51 (18)	
	Stress avoidance	36 (12.7)	
	Changing medication	28 (9.9)	

**Table 5:** Using information and communication technology (ICT) for inflammatory bowel disease (IBD) self-management (questions 18-20; n=724).

		Participants, n (%)	ATI^a^ score						
			1	2	3	4	5	6	7
**Uses ICT for IBD self-management**									
	Total	724 (100)	33	157	46	190	109	85	102
	Yes	405 (55.9)	20	96	19	83	35	17	27
	No	300 (41.4)	12	56	26	100	70	66	74
	I don’t know	18 (2.5)	1	3	0	7	4	2	1
	Not answered	1 (0.1)	0	2	1	0	0	0	0
**ICT hardware use**									
	Smartphone	116^b^ (16)	—^c^	—	—	—	—	—	—
	Wearable tracker	108 (14.9)	—	—	—	—	—	—	—
	Smartwatch	99 (13.6)	—	—	—	—	—	—	—
	Smart domotics	11 (1.5)	—	—	—	—	—	—	—
	No ICT fits me	119 (16.4)	—	—	—	—	—	—	—
**ICT software use**									
	MyIBDcoach	99 (13.6)	—	—	—	—	—	—	—
	IBD information sites	47 (6.5)	—	—	—	—	—	—	—
	Hospital-provided apps	46 (6.4)	—	—	—	—	—	—	—
	Generic health apps	32 (4.4)	—	—	—	—	—	—	—

^a^ATI: Affinity for Technology Interaction.

^b^It is important to bear in mind that this does not mean that only 116 participants use a smartphone; participants with low ATI scores are more likely to talk about their ICT use for IBD self-management in more abstract terms, mentioning “smartphone” or “apps and websites,” whereas participants with high ATI scores are more likely to not mention the smartphone but only mention the specific apps they are using for the self-management of their condition (eg, Strava, an activity tracking app, or DEARhealth, an IBD self-management app).

^c^Not applicable.

### Saturation

The coding process took place in batches of results from 25 participants and resulted in 165 codes. In the first batch, we used 137 unique codes; the second batch yielded another 10 unique codes, the third batch another 14 codes, the fourth batch another 7 codes, and the fifth batch another 9 codes. After these 5 batches, new codes never exceeded a threshold value of 2.5%, indicating full code saturation after 125 participants. Furthermore, all cells in the ENA analyses exceeded the rule-of-thumb threshold of 40 participants.

### Using ICT for IBD Self-Management

#### Quantitative Results

A small majority of the participants (405/724, 55.9%) reported currently using ICT for the self-management of their condition. This use had no significant correlations with any other determinant (eg, age, sex, education, and condition type), except with the score on the self-reported Affinity for Technology Interaction (ATI) questions. Participants who reported using ICT for self-management of IBD had a higher average ATI score (mean 4.61, SD 1.8031) than those who reported using no ICT (*µ*=3.57, *r*_722_=0.2937, SE 0.03979; *P*<.001). To assess which factors best predicted the use of ICT for the self-management of IBD, we performed a binomial general linear model analysis. This analysis revealed a significant contribution of the ATI score to ICT use for self-management; again, participants with higher ATI scores had a greater chance of also using ICT (estimate=0.50323, SE 0.08592, *z*=5.857; *P*<.001); furthermore, there were weakly significant (*P*<.05) contributions of years since diagnosis, with participants diagnosed for >25 years having a slightly smaller chance of using ICT for self-management (estimate=–1.49372, SE 0.69406, *z*=–2.152; *P=*.03); and perceived influence on exacerbations, with perceived influence having a small positive effect on the chance of using ICT (estimate=0.84647, SE 0.32910, *z*=2.572; *P*=.01). Table 5 presents an overview of the results from the questions related to using technology for the self-management of IBD.

#### Qualitative Results: Using ICT for Self-Management (Thematic Analysis)

Thematic analysis made it possible to construct 3 main themes in perceived use cases for ICT in IBD self-management: *learning about your condition*, *living with your condition*, and *emotional and peer support*. First, participants stated that ICT could be useful in learning about their condition. Many participants mentioned using ICT to document various aspects of their condition, such as symptom occurrence and severity, as well as documenting the occurrence and impact of the perceived determinants of their condition, such as nutrition, stress, and sleep. Some participants used this information for self-diagnosis or to prognosticate about their condition. Participants also mentioned using ICT to find information about their condition, and some participants expressed a wish that the medical profession should make greater use of ICT:

[I use the] digital patient contact system to keep sight on appointments, to communicate with my care team, and for insight in [blood] test results, so I know quickly if everything is under control and I can remain in the driver’s seat.

Participant 76, male, aged 35 years, CD

Mapping your own values, so you know if anything deviates. The standards in medical measurement values do not apply to everyone. What would for most people be a regular blood pressure or body temperature would for me be a cause for alarm, so I need to know my own values. If I get a flare-up, it is practical to be able to look back at my values and identify the onset. I notice, for instance in hospital admissions, that they rely on numbers from theory too much. They should listen to the patient more.

Participant 113, female, aged 48 years, CD

The stool app provides a nice clear overview. The hospital app is supposed to make communication with the hospital easier and more accessible, but in my experience this does not work. I still need to call them and wait for them to call back. IBDream does not give me added value, but the hospital prefers it.

Participant 266, female, aged 35 years, CD

A second theme concerned the practical aspects of living with the condition. Participants mentioned using activity tracking and other forms of monitoring to ensure that they maintained a healthy lifestyle to better enable them to deal with their condition and change their behavior (*directive*/*behavior change*). Participants also mentioned using ICT to connect with their care professionals, which reduced the burden their medical care entails, for instance, by opting for online appointments instead of making in-person visits and asking questions through apps and emails. Finally, participants mentioned using apps to find nearby toilets:

Because [the app] makes it more transparent how active I am and how much progress I make, I regain confidence in my body.

Participant 27, male, aged 36 years, UC

An immediate overview of everything I need for a healthy life. For instance, Fitbit’s recovery score. It measures sleep and activity and calculates a daily recovery score. If I have a very bad day, the app provides me with the confirmation I need to take a step back.

Participant 373, female, aged 44 years, UC

When I was ill, I could make connections between flare-ups, heart beats, and a deteriorating cardiovascular condition. My activity went down to 0 immediately. I could not exert myself without immediately having to run for the toilet. Since I have been capable to do sport again, I like to keep track of my progress. At work, I take between ten thousand and eighteen thousand steps per day.

Participant 218, male, aged 30 years, UC

I like to know if I have been active enough each day, that’s why I use the activity tracker—I think being active helps me. Also, I feel exhausted quickly because of my condition, so I like to be able to check how my sleep was.

Participant 527, female, aged 20 years, CD

A third theme concerned using ICT for emotional and peer support. Participants mentioned using ICT to connect with, and find out about, others with their condition, as well as to stay calm and detect stress:

Social media: by following others with IBD [or other autoimmune conditions] I feel less alone in my process. I have dear friends, but they will never completely understand what it means to have a chronic condition.

Participant 249, female, aged 41 years, UC

I like to read about situations that I recognize, so I know I am not the only one. And sometimes tell my story, get support, advice, tips.

Participant 526, male, aged 66 years, CD

It helps me find rest, so I know everything is taken care of and I don’t get stressed, for example, about not getting my medicine in time.

Participant 88, male, aged 57 years, UC

#### ENA Results: Using ICT for Self-Management

The main findings in this analysis were as follows:

- All participants saw ICT as a method to connect to health care professionals and find information.
- Participants who were already using ICT also did so to document their condition and monitor physical activity.
- Participants who were not using ICT saw ICT as a tool for diagnosis, behavior change support, and finding toilets.

**Figure 1:**
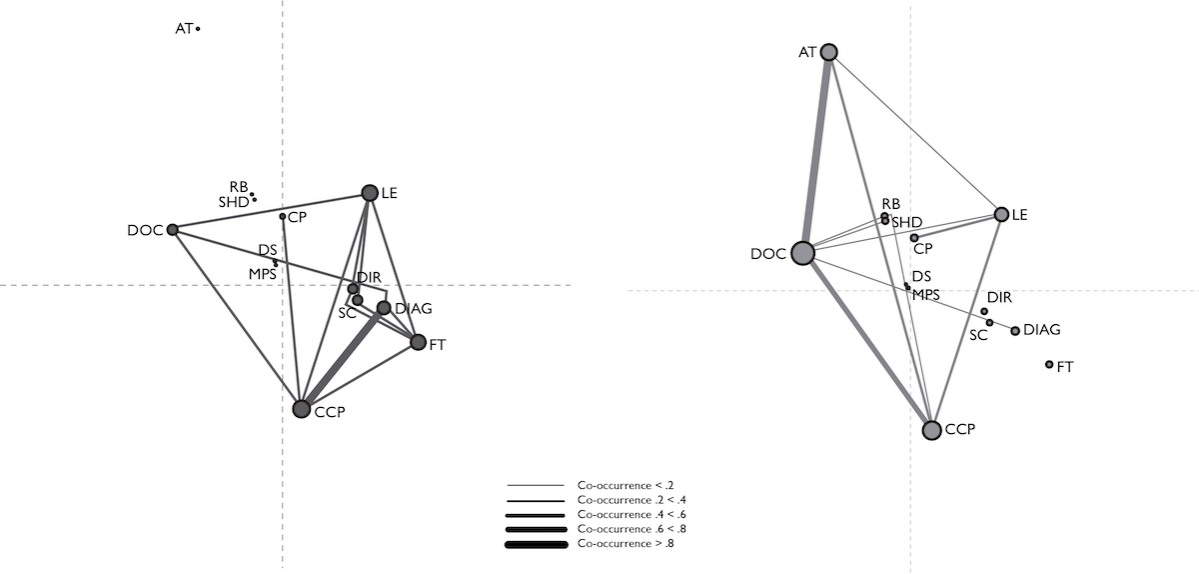
Epistemic network analysis results for all codes from the "perceived use cases for information and communication technology" (ICT) subgroup. (A) The network for the response No (ie, participants who reported not using ICT for inflammatory bowel disease [IBD] self-management; n=300). (B) The network for the response Yes (ie, participants who reported using ICT for IBD self-management; n=405). The abbreviations DIAG (diagnosing condition), DOC (documenting condition), LE (learning and finding information) and MPS (medical profession should do more) apply to the theme "learning about condition." The abbreviations AT (activity tracking or keeping active), CCP (connecting with care professionals), DIR (directive tracking or behavior change), FT (finding toilets), RB (reducing the burden of medical care), and SHD (supporting a healthy diet) apply to the theme "living with condition." The abbreviations CP (connecting with peers), DS (detecting stress), and SC (staying calm) apply to the theme "emotional and social support.".

To find out whether participants who were using ICT for IBD self-management differed from those who were not using ICT regarding their perceived use cases for ICT, we performed an ENA using all codes from the *perceived use cases* subgroup as codes, with scores on the binomial variable *using technology for self-management* plus participant numbers as units, and all utterances from the questionnaires as conversations, with a moving stanza window of 6 utterances. Figure 1A shows the network for the response *no* (ie, participants who reported not using ICT for self-management, n=300), and Figure 1B shows the network for the response *yes* (ie, participants who reported using ICT for self-management, n=405).

There are 3 sources of information in each graph: node size, node position, and node connections. The node size refers to how often the depicted code co-occurs with any other code, which can be seen as a measure of relative importance of the code. In Figure 1, *documenting condition*, *connecting with care professionals*, and *activity tracking/keeping active* are the codes with the most connections overall for participants who were using ICT for self-management (Figure 1B), whereas *connection with care professionals*, *learning/finding information*, *diagnosing condition*, and *finding toilets* are the codes with the most connections for participants who were not using ICT for self-management (Figure 1A). When it comes to node position, nodes at a central position in the graph have a smaller contribution to the explained variance in the model than nodes in more peripheral positions. Again, the most important contributors are *documenting condition* and *activity tracking/keeping active* for people who were using ICT; and *connecting with care professionals*, *diagnosing condition*, *learning/finding information*, and *finding toilets* for people who were not using ICT. Finally, connection strength, depicted as the thickness of the lines between the nodes, shows the relative frequency of the co-occurrence of codes. In participants who were using ICT for self-management, the strongest connections are between *documenting* *condition* and *connecting with care professionals* and between *documenting* *condition* and *activity tracking/keeping active*. *Finding toilets*, *staying calm*, *detecting stress*, and *directive tracking/behavior change* do not play a role in the network.

For participants who were not using ICT, the strongest connection is between *documenting* *condition* and *diagnosing condition*. *Activity tracking/keeping active*, *reducing the burden of medical care*, *supporting a healthy diet*, and *medical professionals should make greater use of ICT* (eg, participants expressing an opinion that their medical professional should put in more effort) do not play a role.

### Using the Smart Toilet Seat

#### Quantitative Results

A majority of the participants stated that they would like to try out the smart toilet seat described in the questionnaire, with 59.7% (432/724) answering *yes*, 15.9% (115/724) answering *no*, and 23.8% (172/724) answering *I don’t know* to the question whether they would want to use it. When asked whether they thought the smart toilet seat would help them manage their condition, of the 724 participants, 349 (48.2%) answered *yes*, 125 (17.3%) answered *no*, and 244 (33.7%) answered *I don’t know*. Wanting to use the smart toilet seat correlated significantly with age group membership, with the older age groups more reluctant to use the smart toilet seat than the younger age groups (*r*_718_=–0.05512, SE 0.04477; *P*<.001) and those with a lower ATI score more reluctant to use the smart toilet seat (*r*_718_=–0.09369, SE 0.04179; *P*<.001). We performed a binomial general linear model analysis to assess what determinants contribute to predicting whether participants would want to use the smart toilet seat. Responding *sometimes* on *perceived ability to predict exacerbations* was the only significant contributor to *expressed desire for smart toilet seat use* (estimate=1.54390, SE 0.50561, *z*=3.054; *P*=.002).

#### Qualitative Results: Smart Toilet Seat Use Cases (Thematic Analysis)

Participants mentioned a diverse range of use cases they envision for the smart toilet seat. Again, we could construct 3 themes: *learning about the condition*, *living with the condition*, and *negative use cases*. In the first theme, *learning about the condition*, documenting symptoms, perceived determinants, and the effects of the condition again play a large role. Similarly, participants saw the smart toilet seat as an instrument to support diagnosis, with many participants mentioning specific diagnostic use cases such as measuring stool composition and urine composition as well as inflammatory markers such as fecal calprotectin. However, when thinking of use cases for the smart toilet seat, participants also mentioned use cases that did not come up previously when discussing the use cases of ICT in general, such as automatically and unobtrusively signaling significant changes in their condition (eg, the onset of an exacerbation):

It would be nice if inflammation markers in stool is available sooner, so I can take action quicker without being confined to my house immediately. Especially the inflammation markers in stool. This also makes it easier if certain nutrients affect this, and sound the alarm if inflammation markers go up without any direct increase in symptoms.

Participant 59, female, aged 44 years, UC

If it were possible to figure out where exhaustion comes from and maybe tackle the causes, or determine an energy level so I can anticipate that, I would be very interested. That would encourage me to act when I see inflammations, let me find out about bad or one-sided nutrition or moisture balance, a lack of certain bacteria, or stool composition.

Participant 103, female, aged 40 years, CD

I think it’s mostly about taking away insecurities about my condition. Are my values normal or not? Do I need to contact my specialist or GP [general practitioner] yet? I would like to reduce that insecurity.

Participant 322, female, aged 63 years, CD

A second theme is *living with the condition*, which concerns use cases aimed at reducing the burden of medical care and informing behavioral change:

Predicting flare-ups and providing tips and instructions that fit my current situation [activity and body monitoring] and personal healthy daily schedule accordingly. Maybe even connect to my diary or advise me to cancel a meeting because of deteriorations. I think that early signaling and implementation of the right protective measures, a flare-up can be reduced, delayed, or even prevented. But it is hard to recognize the right signals and give priority to the right actions within the mess of everyday life. A machine or app can measure much more effectively and provide programmed actions based on measured values. That relieves me as human from the daily hassle of dealing with my condition, which would take away a lot of stress. It might even, in the long run, reduce fatigue and give more energy.

Participant 512, male, aged 37 years, UC

Finally, participants also mentioned a range of *negative use cases*. Of the 724 participants, 112 (15.5%) stated that they did not feel any need for the smart toilet seat, would rather not use it, or did not know whether the toilet would have any benefits for them. Some of the participants said that they could not use the toilet because of a stoma or other physical condition:

I think, right now, for me it would be “overkill” to use this, because I am doing fine on my current medication, for now. I would rather do my own tests, when I feel the need.

Participant 305, female, aged 57 years, UC

That too much relies on the smart measurements. If you know your own body well, you will feel that things are off, and you lose that knowledge when you solely rely on measurements.

Participant 165, female, aged 62 years, UC

I would rather live without technology. What will be, will be.

Participant 332, male, aged 67 years, CD

#### ENA Results: Smart Toilet Seat Use Cases

The main findings in this analysis were as follows:

- We compared codes occurring in 4 groups of participants: those who were not using ICT for self-management and would not like to try the smart toilet seat (N-N), those who were using ICT for self-management and would not like to try the smart toilet seat (Y-N), those who were not using ICT for self-management and would like to try the smart toilet seat (N-Y), and those who were using ICT for self-management and would like to try the smart toilet seat (Y-Y).
- Documenting their condition was seen as an important use case for the smart toilet seat by all groups, apart from group N-N.
- Group N-N often mentioned *no need for the smart toilet seat, would rather not use*, and *cannot use because of stoma* (as a reason).
- Group N-Y often mentioned a combination of *signaling changes and directive tracking* (ie, receiving an alarm when something is amiss and being given advice on what to do).
  *Measuring inflammation markers, using for diagnosis* (in combination with *signaling changes*), and *measuring stool composition* also occurred often.
- Group Y-N members often mentioned that they did not know what to use the toilet for and that current advice sufficed; when discussing use cases, they most often mentioned documenting their condition, combined with reducing the burden of medical care and measuring calprotectin.
- Group Y-Y most often mentioned *documenting for (self-)diagnosis*, with a strong connection with *signaling changes*. Furthermore, the group members often mentioned *measuring calprotectin, stool composition, and inflammation markers*, as well as *reducing the burden of medical care*.

**Figure 2:**
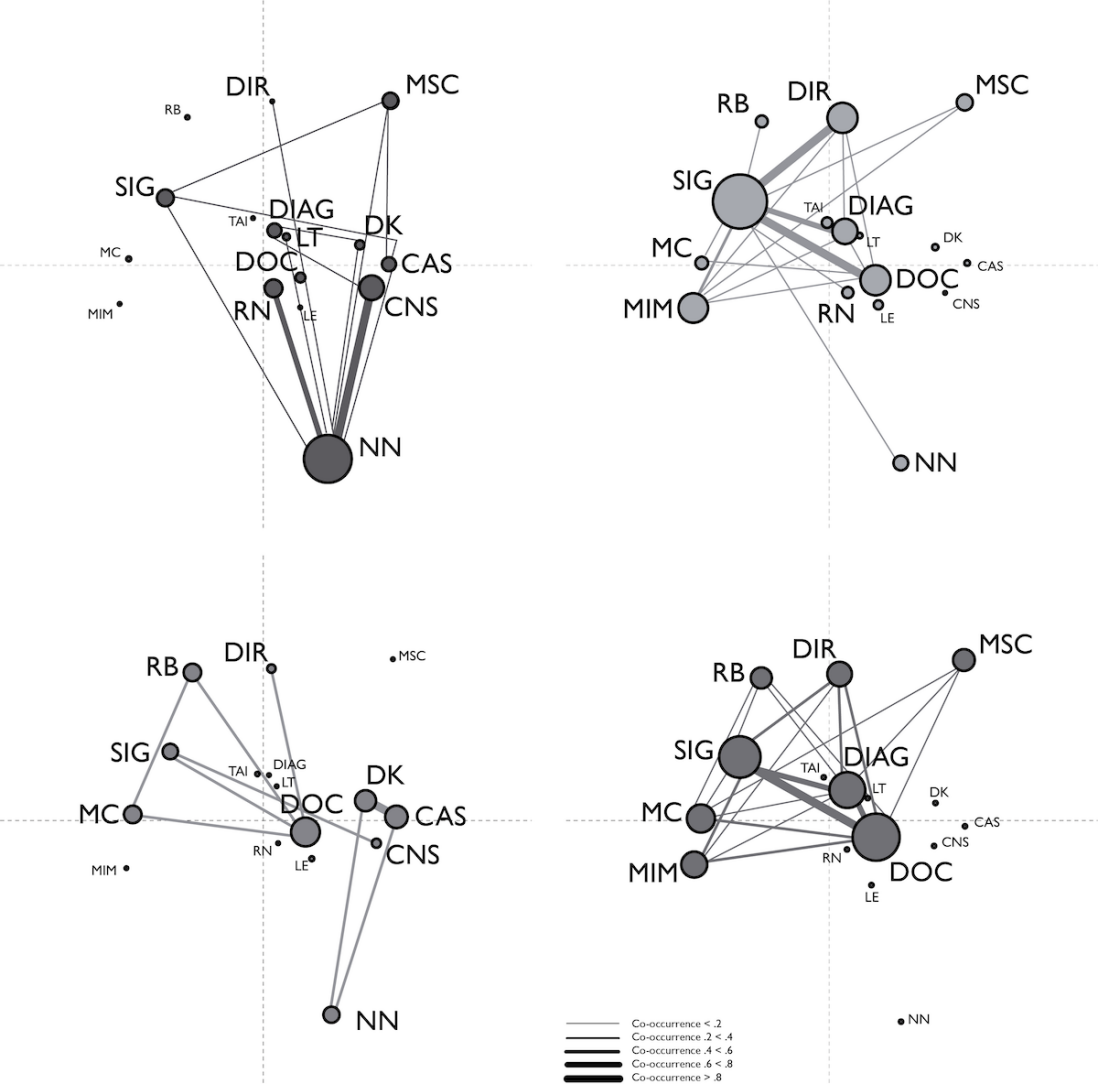
Epistemic network analysis results for all codes from the "perceived use cases for the smart toilet seat subgroup." (A) Code network for participants who were not using information and communication technology (ICT) for self-management and would not want to use the smart toilet seat (N-N). (B) Code network for participants who were not using ICT for self-management and would want to use the smart toilet seat (N-Y). (C) Code network for participants who were using ICT for self-management and would not want to use the smart toilet seat (Y-N). (D) Code network for participants who were using ICT for self-management and would want to use the smart toilet seat (Y-Y). The abbreviations DIAG (diagnosing condition), DOC (documenting condition), LE (learning or finding information), MC (measuring calprotectin), MIM (measuring inflammation markers), MSC (measuring stool composition), SIG (signaling changes), and TAI (tailoring treatment to my situation) apply to the theme "learning about condition." The abbreviations DIR (directive tracking or behavior change), LT (love of technology), and RB (reducing the burden of medical care) apply to the theme "living with condition." The abbreviations CAS (current advice suffices), CNS (cannot because of stoma), DK (don’t know), NN (no need), and RN (rather not) apply to the theme "negative use cases.".

To find out whether participants who were using ICT for IBD self-management differed from those who were not using ICT regarding their perceived use cases for the smart toilet seat, and whether, within these 2 groups, participants who said they would like to use the smart toilet seat differed from those who said they did not want to use the toilet, we performed an ENA using all codes from the *smart toilet seat perceived use cases* subgroup as codes and all utterances from the questionnaires as conversations, with a moving stanza window of 6 utterances. In this ENA, we compared scores on 2 variables (*using ICT for IBD self-management* and *desire to use the smart toilet seat*) to compose four units: (1) those who were not using ICT for IBD self-management and did not want to use the smart toilet seat (N-N, n=70), (2) those who were not using ICT for IBD self-management but were interested in using the toilet (N-Y, n=141), (3) those who were using ICT for IBD self-management but did not want to use the smart toilet seat (Y-N, n=42), and (4) those who were using ICT for IBD self-management and wanted to use the smart toilet seat (Y-Y, n=282). In this analysis, participants who answered *I don’t know whether I want to use the smart toilet seat* (those who were not using ICT for IBD self-management: n=86, those who were using ICT for IBD self-management: n=79) were left out. Figures 2A-2D show the networks for the 4 groups (N-N, N-Y, Y-N, and Y-Y*)*.

Again, information can be derived from node sizes, node positions, and connections between nodes. The node size refers to how often the depicted code co-occurs with any other code, which can be seen as a measure of the relative importance of the code; the position of the node shows the code’s relative contribution to the differences between units, and the connection strength between codes shows the relative frequency of co-occurrence of codes. In the N-N group, the codes *no need*, *rather not*, and *cannot use because of stoma* have the most overall connections, with the code *no need* also making the largest contribution to differences with the other groups. When it comes to connections, the combination of *no need* with *rather not* and with *cannot use because of stoma* are the most prominent. Some nodes remain unconnected and play no role for this group: *reducing the burden of medical care*, *tailoring treatment to my situation*, *measuring calprotectin*, *measuring inflammation markers*, and *learning/finding information*. In the N-Y group, the code *signaling changes* has the most connections overall, with further well-connected nodes being *directive tracking/behavior change*, *measuring inflammation markers*, *documenting condition*, and *diagnosing condition*. Nodes with the most contribution to differences with other groups are *signaling changes* and *directive tracking/behavior change*. The nodes with the strongest connections are the combinations *signaling changes*+*directive tracking/behavior change* and *signaling changes+documenting condition*. Nodes with no connections are *current advice suffices*, *don’t know*, and *cannot because of stoma*. In the Y-N group, there are no nodes that have a distinctively larger size than others, with *documenting condition* being the largest node. Similarly, connections between nodes for this group are relatively weak, with the exception of the connection between the nodes *don’t know* and *current advice suffices*. Finally, in the Y-Y group, the nodes corresponding with the codes *documenting condition*, *signaling changes*, and *diagnosing* *condition* are the largest, with *signaling changes* and specific cases of diagnostic tracking (*measuring stool composition*, *measuring calprotectin*, and *measuring inflammation markers*) contributing the most to the differences with the other groups. When it comes to inflammation markers, this group mentioned the specific marker (calprotectin) more often than the general term (inflammation markers), whereas the N-Y group mentioned the general term more than the specific marker. When it comes to connection strength, the combinations *documenting condition*+*signaling changes* and *signaling changes*+*diagnosing condition* are the strongest.

#### Qualitative Results

##### Concerns and Data Agency: Summative Content Analysis

Regarding data agency, a majority of the participants (418/724, 57.7%) stated that they would like their data to be always available to themselves (eg, through a smartphone app or dashboard); 17% (123/724) wanted to receive reports only when something happened that necessitated action; 8% (58/724) would not want constant access to data, but they would want regular summaries of key information; and 3.2% (23/724) wanted reports to go directly to their health care provider only.

##### Concerns and Data Agency: Thematic Analysis

Besides perceived use cases, participants also mentioned perceived concerns about using the smart toilet seat. For these concerns, 4 major themes could be constructed: *privacy and data agency*, *practical concerns*, *concerns about dealing with the condition*, and *validity*. First, many participants cited *privacy and data agency* (ie, who has control over accessibility to the data) as a major concern:

Privacy issues; my data can end up with the wrong persons or companies, or worse: get connected—not anonymously—to my name and other personal data.

Participant 13, female, aged 53 years, CD

Evidence of my disease in facts. That is a relief, because you do not have to constantly think about how you are doing. The question that remains, though, is who else can see these data. You don’t want to share these data with just anybody, maybe even with nobody at all.

Participant 242, male, aged 62 years, CD

A second theme involves practical concerns. The cost of purchasing and having a smart toilet seat, how the toilet can distinguish between different users, technological issues, breakdowns, hassle, and hygienic concerns all played a role for many participants:

It will probably be costly, that makes sense, but it is a barrier.

Participant 178, female, aged 40 years, CD

How can the smart toilet seat figure out who is on the toilet? It is important that different people can use the toilet without mixing up data.

Participant 260, female, aged 60 years, UC

The whole digital business, it hardly ever works for me, or only partially.

Participant 294, male, aged 78 years, CD

A third theme involves participants’ concerns about how a smart toilet seat will affect living and dealing with their condition. Some participants mentioned a fear of unnecessary worries; they do not want a constant reminder of their illness and want to avoid overmedicalization of their condition or the replacement of real-life contact with health care professionals by machines:

It only leads to more unrest, I think. I don’t want to be thinking about my disease all day, I want to think about being healthy.

Participant 16, female, aged 52 years, CD

I think that every time an abnormal measurement comes up, I would get very stressed, and that is a problem if your gut responds to stress strongly.

Participant 320, female, aged 63 years, CD

That in the end, a doctor’s visit is deemed superfluous by health insurance. A patient is more than their excrement.

Participant 289, female, aged 72 years, CD

A final, fourth theme involves concerns about the *validity* of the smart toilet seat measurements. This theme encompasses concerns about the validity and reliability of measurements, especially for participants’ specific situation, and unnecessary data generation:

I think this will not help me, because blood and excrement tests have never shown an inflammation with me.

Participant 337, female, aged 59 years, CD

I don’t see how the information gathered here could help me manage my condition.

Participant 538, female, aged 42 years, CD

#### ENA Results: Smart Toilet Seat Use Concerns

The main findings in this analysis were as follows:

- A shared concern for all groups is privacy.
- People who were not using ICT for self-management (N-N and N-Y groups) often mentioned fear that the toilet would not work, unnecessary worries, hassle, and technical issues.
- People who would not like to try the smart toilet seat (N-N and Y-N groups) mentioned constant reminders of their illness and overmedicalization of their condition as concerns, as well as the reliability and validity of the smart toilet seat’s measurements.
- The N-N group also often mentioned unnecessary data generation and the replacement of health care professionals by technology as potential downsides.
- The Y-N group also often mentioned cost, data agency, and the replacement of health care professionals by technology as potential downsides.
- The N-Y group also mentioned concerns about whether others could use the toilet without messing up the measurements and data agency as potential downsides.
- The Y-Y group had mostly practical concerns: potential issues with the technology, others using the smart toilet seat, cost, and data agency.

**Figure 3:**
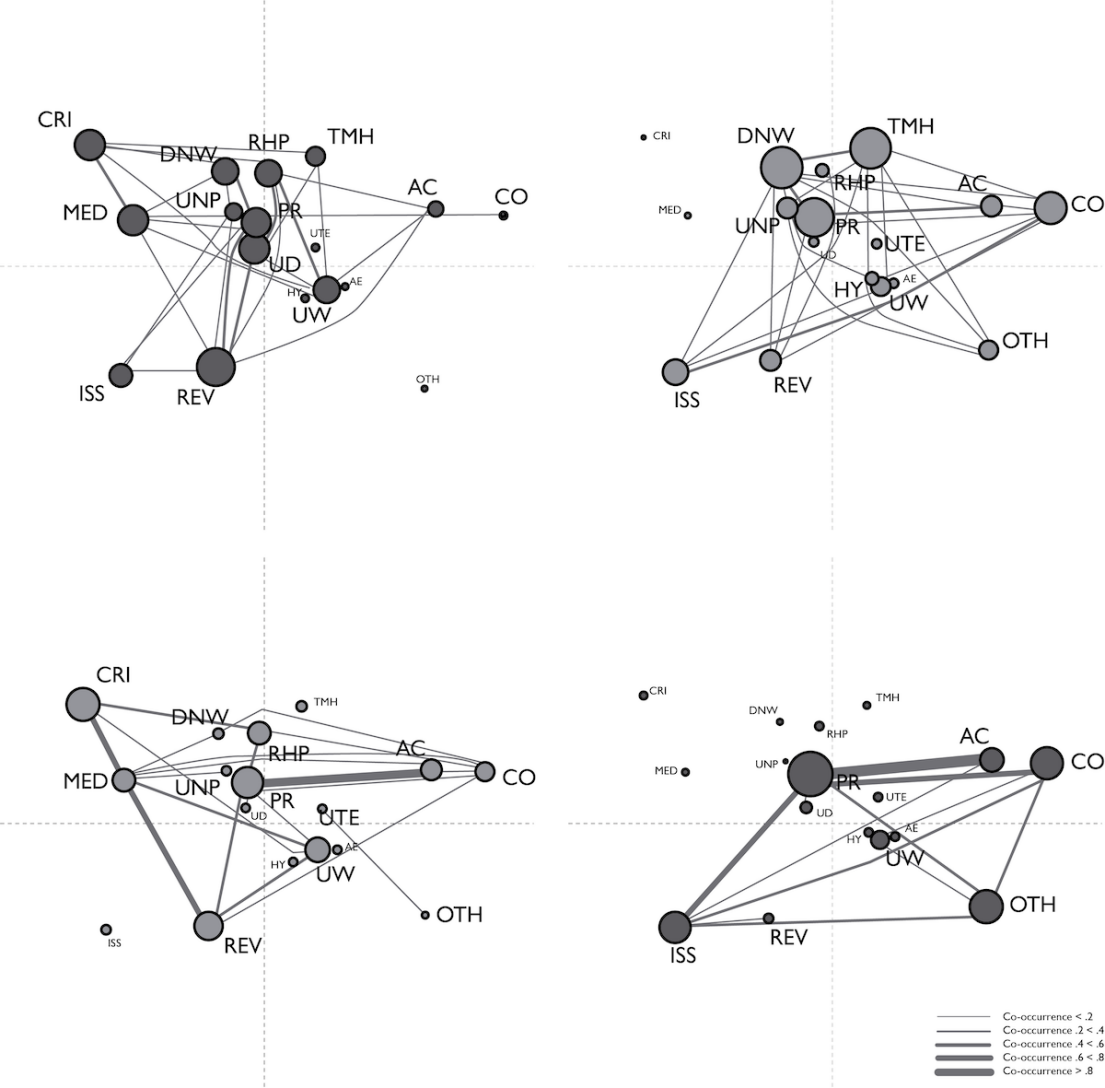
Epistemic network analysis results for all codes from the "concerns about the smart toilet seat" subgroup. (A) Code network for participants who were not using information and communication technology (ICT) for self-management and would not want to use the smart toilet seat (N-N). (B) Code network for participants who were not using ICT for self-management and would want to use the smart toilet seat (N-Y). (C) Code network for participants who were using ICT for self-management and would not want to use the smart toilet seat (Y-N). (D) Code network for participants who were using ICT for self-management and would want to use the smart toilet seat (Y-Y). The abbreviations AC (agency control) and PR (privacy) apply to the theme privacy and data agency. The abbreviations AE (aesthetics and design), CO (cost), HY (hygiene), ISS (technical issues), OTH (others using the toilet), TMH (too much hassle), and UNP (unpleasant) apply to the theme "practical concerns." The abbreviations CRI (constant reminder of illness), MED (overmedicalization of condition), RHP (replacement of health care professionals by technology), and UW (unnecessary worries) apply to the theme "concerns about dealing with the condition." The abbreviations REV (reliability and validity), DNW (won’t work [for me]), and UD (unnecessary data generation) apply to the theme "validity.".

To find out whether participants who were using ICT for IBD self-management differed from those who were not using ICT regarding their concerns about using the smart toilet seat, and whether, within these 2 groups, participants who said that they would like to use the smart toilet seat differed from those who said they did not want to use the toilet, we performed an ENA using all codes from the *smart toilet seat concerns* subgroup as codes and all utterances from the questionnaires as conversations, with a moving stanza window of 6 utterances. In this ENA, as before, we compared scores on *using ICT for IBD self-management* and *desire to use the smart toilet seat* to compose four units: (1) N-N, n=70; (2) N-Y, n=141; (3) Y-N, n=42; and (4) Y-Y, n=82 (Figures 3A-3D, respectively).

As before, information can be derived from node sizes, node positions, and connections between nodes. The ENA shows that *privacy* was a concern for every participant, and therefore the position of the node shows that it does not contribute to distinguishing between the groups. However, the connections between *privacy* and other nodes differ for the 4 participant groups. Apart from *privacy*, when it comes to node size, all codes from the theme *concerns about dealing with the condition* play an important role in the N-N group: *constant reminder of illness*, *overmedicalization of condition*, *unnecessary worries*, and *replacement of health care professionals by technology*. Furthermore, codes from the theme *validity*, such as *won’t work (for me)*, *reliability and validity*, and *unnecessary data generation*, are most notable. No edges between nodes stand out particularly, although the combination of *constant reminder of illness* with *overmedicalization of condition* occurs more often than others. For the position of nodes, *constant reminder of illness*, *overmedicalization of condition*, and *reliability and validity* contribute the most to distinguishing this group from others. Not connected are nodes from the *practical concerns* theme, such as *others using the toilet*, *hygiene*, *aesthetics and design*, and *using toilets elsewhere*. In the N-Y group, besides *privacy*, the most notable nodes are *cost*, *issues with technology*, and *too much hassle* from the *practical concerns* theme; and *won’t work (for me)* from the *validity* theme. Nodes without connections are *constant reminder of illness*, *overmedicalization of condition*, *replacement of health care professionals by technology*, *aesthetics and design*, and *unnecessary data* *generation*. No connections between nodes stand out in particular, although the combination of *privacy* with *agency control* and that of *cost* with *issues with technology* have stronger connections than others. Node position shows that *issues with technology*, *cost*, *won’t work (for me)* and *too much hassle* contribute the most to distinguishing this group from the others. In the group Y-N, *constant reminder of illness*, *overmedicalization of condition*, *unnecessary worries*, and *replacement of health care professionals* *by technology* from the *dealing with condition* theme; *reliability and validity* from the *validity* theme; *privacy* and *agency control* from the *privacy* theme; and *cost* from the *practical concerns* theme are the most prominent. Connections between *constant reminder of illness* and *overmedicalization of condition*, *overmedicalization of condition* and *reliability and validity*, and *privacy* and *agency control* stand out. As for node position, *constant reminder of illness*, *overmedicalization of condition*, *reliability and validity*, *agency control*, and *cost* contribute the most to distinguishing this group from other groups. *Issues with technology*, *too much hassle*, *unnecessary data* *generation*, *hygiene*, and *aesthetics and design* have no connections with other nodes. Finally, in the Y-Y group, the node sizes of *privacy* and *agency control* from the *privacy* theme as well as *cost*, *others using the toilet*, and *issues with technology* from the *practical concerns* theme stand out. There are strong connections between *privacy* and *agency control*, *privacy* and *issues with technology*, and *privacy* and *cost*. Node position shows that *issues with technology*, *cost*, *others using the toilet*, and *agency control* contribute the most to distinguishing this group from others. Nodes from the *concerns about* *dealing with the condition* theme (eg, *constant reminder of illness*, *overmedicalization of condition*, and *replacement of health care professionals by technology*) and those from the *validity* theme (eg, *won’t work* [*for me*]), have no connections.

## Discussion

### Principal Findings

In this study, we aimed to investigate the perspectives of people with IBD regarding the use of ICT for self-management of their condition. To do so, we constructed a questionnaire and analyzed the responses provided by 724 participants. Through this analysis, we sought to identify the use cases and barriers perceived by individuals with IBD in adopting ICT-based solutions for managing their health condition. Among the participants in the study, more than half (405/724, 55.9%) were already using various forms of ICT for IBD self-management. The primary factor influencing their current use of ICT was their affinity for interacting with technology. The questionnaire also shed light on self-management needs and skills among persons with IBD. A large majority (never: 86/283, 30.6%; sometimes: 135/283, 48%) could often not predict exacerbations, and although many participants (203/283, 71.7%) thought they knew what caused exacerbations, most (203/283, 71.7%) did not know how to influence them. Participants indicated needs for social support (105/283, 37.1%), information support (81/283, 28.6%), and resilience and optimism (64/283, 22.6%); their current strategies were sleep and relaxation (65/283, 22.9%), avoiding trigger foods and maintaining a healthy diet (51/283, 18%), stress avoidance (36/283, 12.7%), and changes in medication (28/283, 9.9%).

Through ENA, distinct differences emerged between individuals who were using ICT and those who were not using ICT, particularly regarding their perceived use cases. Participants who were using ICT viewed it as a means to document their condition, monitor physical activity, and establish connections with health care professionals to share their insights. By contrast, the individuals not using ICT regarded it as a tool to document their condition, aiming to support professional diagnosis.

Upon considering the perceived cases and concerns related to using a smart toilet seat as an example of ICT for IBD self-management, the sample population can be categorized into 4 distinct groups. First, those who were not using ICT for the self-management of their condition and had no interest in using the smart toilet seat (N-N), accounted for 13.1% (70/535) of the respondents who provided a response to the question regarding their interest in the technology. Second, those who were not using ICT but expressed a desire to use the smart toilet seat (N-Y) constituted 26.4% (141/535) of the sample. Third, individuals who were using ICT for IBD self-management but were not interested in using the smart toilet seat (Y-N) represented 7.9% (42/535) of the participants. Finally, the majority of the respondents (282/535, 52.7%) were individuals who were using ICT for IBD self-management and expressed an interest in using the smart toilet seat (Y-Y).

These groups exhibited distinct variations in perceived use cases and concerns associated with the adoption of a smart toilet seat for IBD self-management. The N-N and Y-N groups lacked clear use cases for the smart toilet seat. The N-Y group members perceived the smart toilet seat as a means to identify detrimental changes in their condition, offering directions for action, and documenting information for health care professionals’ diagnoses. By contrast, although the Y-Y group members also viewed the smart toilet seat as a means to signal negative changes in their condition, they stood apart from the N-Y group in that they stated their intention to document these changes for their own self-management and decision-making.

Similarly, these groups expressed different concerns regarding the use of the smart toilet seat. The N-N group members expressed worries about the potential impact on daily life with their condition, including constant reminders of their health status. In addition, practical concerns, such as privacy and the accuracy of the measurements, were also prominent for this group. The Y-N group mainly raised concerns related to data agency and privacy issues. The N-Y group emphasized privacy concerns and practical aspects such as the potential hassle and associated costs. Finally, the Y-Y group members, again, shared concerns about privacy and data agency, along with the apprehension of others using the smart toilet seat, potentially compromising their personal information and health data.

This research provides new insights about patient perspectives on using ICT for IBD self-management, which are currently not available in the literature. Some of the results confirm earlier work with more general participant groups [21], in which signaling and documenting also appear as important use cases, with privacy and data agency emerging as major concerns. However, this study also offers valuable insights into effectively addressing the needs of individuals who exhibit an inclination toward technology interaction but remain hesitant to embrace proposed interventions. Providing assurance regarding privacy and data agency becomes essential in catering to this particular group.

The research shows that there is also a group of people who have low affinity for technology and would therefore not want to use an ICT-based intervention. They believed that such an intervention would be too much of a hassle and that it was not for them. These opinions need to be respected. However, a substantial number of participants in this group (N-N) talked about wanting to avoid constant reminders of their condition and overmedicalization of their condition. This may be a sign of detrimental coping by hiding or denying their condition [42,51], which may impact IBD coping and self-management negatively [42,43]. It is imperative to conduct further research in this area to gain a deeper understanding of the implications of these coping strategies on IBD coping and self-management. By exploring these aspects more comprehensively, we can develop tailored approaches and interventions that better align with the needs and preferences of individuals with low technology affinity while promoting effective disease management and self-care.

When it comes to other results from this study, we see that there is a participant bias in that more female individuals than male individuals participated. Although the participant rate for female and male individuals (60% and 40%, respectively) reflects sex differences in the occurrence of IBD in the Dutch population [52,53], the occurrence of CD and UC in the sample in this paper (approximately 55% and 45%, respectively) differs from population data (approximately 40% and 60%, respectively) [52,53]. This may be caused by the aforementioned participation bias, a recurring pattern in questionnaire research [54,55]. Similarly, the literature [54,55] on questionnaire research indicates that it is relatively common for older participants to be overrepresented compared to population numbers. However, in this study, we think this is not problematic because there were sufficient male participants (233/724, 32.2%) to achieve saturation and stratification, as well as adequate representation of people aged <31 years (76) and those aged between 31 and 40 years (109). Furthermore, highly educated participants were overrepresented in the sample. Notably, those with only primary school education, who can be expected to have limited health literacy, were underrepresented. Given the fact that this group often has limited language skills [52] and is therefore less likely to participate in web-based questionnaires, further research is necessary to learn about their needs and barriers.

Finally, when it comes to use cases for ICT for IBD in general, 3 major themes were constructed: *learning about the condition*, *dealing with the condition*, and *connecting with others*. The smart toilet seat only covers the first 2 themes; the third theme is difficult to integrate in this solution because toilet-related topics are often awkward to talk about or even taboo [21]. To fulfil people’s needs around connecting with others, a stand-alone solution would be preferable, especially considering the concerns that people have about privacy and data agency.

### Conclusions and Recommendations

In conclusion, this study provides valuable insights into the perspectives of individuals with IBD on using ICT for self-management. The positive reception of the participants to the smart toilet seat concept suggests the potential for innovative ICT solutions in empowering patients to actively participate in their care. To facilitate wider adoption, it will be critical to address privacy concerns, ensure data security, and establish reliable ICT integration. Furthermore, the results of our research show that one-size-fits-all solutions will likely fail to reach many potential users of technological innovations for disease self-management because they do not connect to their perceived use cases or do not address their concerns. Use cases differ from simply signaling when something is amiss and providing an action perspective to informing personal scientific experiments. Patients’ concerns differ from fear of overmedicalization of their condition and constant reminders of the illness to concerns about cost, privacy, and data agency. This paper not only shows that people differ but also provides evidence that there are possibilities for profiling large groups of potential users with similar needs and barriers. The results of this study and methodologically similar future studies can inform implementation and dissemination strategies for technological innovations. Future research and development should not only focus on refining and validating the technologies to enhance their effectiveness and user acceptance, with extra focus on patients with limited health literacy or low affinity with technology interaction, but also strive to shed greater light on who the user is, what their needs and requirements are, and how their lived experience of their condition informs their use of technology for self-management.

## Appendix

Multimedia Appendix 1 Full questionnaire, translated into English.

Multimedia Appendix 2 Overview of themes, subthemes, codes, and code frequencies for each part of the questionnaire. Part 1: disease characteristics and self-management; part 2: information and communication technology use in self-management; and part 3: the smart toilet seat. Codes from disease characteristics, predictors, perceived causes, self-management strategies, self-management needs, and hardware and software use are derived from the summative content analysis. All other codes are derived from the thematic analysis.

## Abbreviations

ATI: Affinity for Technology Interaction
CCNL: Crohn & Colitis NL
CD: Crohn disease
ENA: epistemic network analysis
IBD: inflammatory bowel disease
ICT: information and communication technology
UC: ulcerative colitis

## Data Availability

The data sets generated and analyzed during this study are available from the corresponding author on reasonable request.
