# Supplementary material for: Investigating Patient Perspectives on Using eHealth Technologies for the Self-Management of Inflammatory Bowel Disease: Mixed Methods Study
Source: J Med Internet Res. 2024 Sep 6;26:e53512. doi: 10.2196/53512 (PMC11415722; doi:10.2196/53512)
Supplement: Multimedia Appendix 1 [file jmir_v26i1e53512_app1.docx]

Questionnaire

Thank you for your willingness to complete this questionnaire. This questionnaire is about how you deal with your bowel disease and how technology can help you. We also propose a possible new technological aid: a 'smart toilet', and ask you what you think about it.

The questionnaire consists of multiple-choice questions, in which you can tick which answer suits you best. There are also a number of open questions, in which you can write down your own answer. It is best to think briefly about your experiences with these questions and then write down your answer. If you are unable or unwilling to answer a question, you can skip it. Completing the entire questionnaire takes 20–25 minutes.

We store all your answers anonymously, meaning without your name or other contact details. Therefore, we will not know which answers are yours. Our research is about people with intestinal diseases in general, so we don't look at your answers separately.

By completing and sending the questionnaire you give permission that your data can be used for research. You can always choose not to participate in the questionnaire while completing it, you can then simply close the questionnaire. Your answers will not be shared with us until you submit your answers at the end of the questionnaire.

This questionnaire has been prepared by OnePlanet Research Center - a multidisciplinary collaboration agreement between Wageningen University & Research (WUR), Radboud University, Radboudumc and nanotechnology innovation center imec - in collaboration with Crohn & Colitis NL.

*The first questions are about your background. We ask these questions because we know that for many people their background influences how they deal with their illness.*

Question 1: What is your age?

Indicate age in years. For example, if you are 39 years old, enter the number 39.

(open input field)

Question 2: What is your sex?

(Choose from Female/Male/Other/Prefer not to say)

Question 3: What education have you had or are you currently following?

(You can indicate your highest level of education)

- Primary education only

- VMBO (also former LBO / Ambachtsschool / Huishoudschool), MAVO, HAVO / VWO up to class 3, MBO-1

- Basic vocational training (MBO-2), vocational training (MBO-3 and 4), HAVO / VWO

- HBO, WO

Question 4: What profession do you have?

(You can select multiple options)

- Incapacity for work, sickness law

- Looking for a job

- Caregiver, family and household duties

- Occupation for which you must have primary education

- Occupation for which you must have completed secondary education

- Profession for which you must have followed a higher or scientific level of education

- I'm not working and I'm not looking

*Illness experience*

*The following questions are about your illness and how you experience it.*

Question 5:

What disease do you have?

- Crohn's disease

- Ulcerative colitis

- IBD without a diagnosis, it is not (yet) clear whether this is Crohn's or ulcerative colitis

- Otherwise, namely...

Question 6:

How long ago were you diagnosed with the disease?

*Dropdown box with the following answers:*

< 1 year ago

1 year ago

2 years ago

3 years ago

4 years ago

5 years ago

6 years ago

7 years ago

8 years ago

9 years ago

10 years ago

11 years ago

12 years ago

13 years ago

14 years ago

15 years ago

16 years ago

17 years ago

18 years ago

19 years ago

20 years ago

21 years ago

22 years ago

23 years ago

24 years ago

Longer than 25 years ago

Question 7:

How does your condition affect your life during a flare-up?

*(Tick what applies to you, multiple answers possible)*

- My condition sometimes makes it difficult for me to perform household tasks independently (e.g. shopping, cleaning, cooking)

- My condition sometimes makes it difficult for me to work

- My condition sometimes makes it difficult for me to maintain social relationships with friends and family

- My condition sometimes makes it difficult for me to maintain a family life

- My condition sometimes makes it difficult for me to do fun things and enjoy myself

- I never have a flare up

Question 8:

When you have a flare up, what do you feel?

*(Tick what applies to you, multiple answers possible)*

- diarrhoea

- needing to go to the toilet frequently

- idle urge

- decreased appetite

- abdominal pain or pain in the lower abdomen

- blood or mucus in the stool

- dry eyes or eye infections

- joint pain

- fistulas or abscesses

- severe fatigue

- fever

- high heart rate

- feelings of depression or anxiety

- otherwise, namely....

- I never have a flare up

Question 9:

Do you have any other conditions besides IBD?

*(Tick what applies to you, multiple answers possible)*

- No

- Joint complaints

- Spondyloarthritis

- Rheumatic disorder

- Bekhterev's disease

- Osteoporosis (bone loss)

- Chronic skin conditions (such as psoriasis, hidradenitis suppurativa)

- Eye disease (e.g. uveitis). We don't mean low vision here

- Oral and dental problems

- PSC (Primary Sclerosing Cholangitis)

- Other liver diseases

- Pancreatitis (disease of the pancreas)

- Thrombosis

- Lung disorders (such as asthma, COPD)

- Blood disorder or blood disorder (e.g. iron deficiency)

- Psychological disorder (such as anxiety or depressive symptoms)

- Otherwise, namely

Question 10:

Have you had a flare-up in the last 12 months?

Yes -> Question 11

No

I do not know

(No or I don't know: -> question 18)

*Coping with your illness during a flare-up*

*The following questions are about how you deal with your illness. Here you can keep your last flare in mind*

Question 11:

When you have a flare-up, do you feel it coming on beforehand?

No -> Question 13

Sometimes

Often

Always

Question 12:

How and when do you feel a flare coming (for example, physical or mental changes, increased temperature, abdominal pain, stress, ...)?

*(Open answer)*

Question 13:

Are there things in life that you know or suspect can trigger a flare-up, such as stress, certain activities, certain foods?

Yes

No -> question 15

Question 14:

Can you tell me what things in life you suspect may trigger a flare-up?

*(Open question)*

Question 15:

Do you feel that you can influence your flare-ups yourself?

Yes

No -> Question 17

Question 16:

How do you think you can influence your flare-ups?

*(Open question)*

Question 17:

What do you think you need to better cope with your illness? For example, think of knowledge, information, skills, support, endurance, optimism, and so on.

*(Open question)*

*Technology*

*The upcoming questions are about technology: technology that we use in everyday life. That is a broad concept, for example devices such as the smartphone (mobile), smart watches or pedometers, but also apps on the telephone and websites. The development of new technology is very fast and we can increasingly use new websites, apps or devices to help us with all kinds of things in life.*

Question 18:

Are you generally interested in new technology?

*Tick what applies to you. You may give multiple answers*

- I like to use new technology

- I'd like to know how everything works

- I like to try new things

- I enjoy investing time in new technology

- I mainly use technology because I have to

- I like to avoid technology

- I want technology to just work without me having to understand anything about it

Question 19:

Do you use technology to cope with your illness? Think of the smartphone, pedometers and other devices, but also websites and apps especially for IBD (IBDream, myIBDcoach)

- Yes -> Question 20

- No -> Question 22

- I don't know -> Question 23

Question 20:

What technology do you use?

*(Open)*

Question 21:

How does this technology help you? --> Question 23

*(Open)*

Question 22:

Why not use technology to cope with your illness?

*(Tick what applies to you, multiple answers possible)*

- I haven't found any apps, wearables, websites that can help me yet

- I did try but it didn't work for me

- I've tried, but I think it's too much of a hassle

- I tried, but I didn't like it

- I prefer not to use these resources

- I find these resources too complicated

- I don't think this helps me anyway

- Otherwise, namely...

Question 23:

Are there aspects of your disease that you would like to use technology (apps, devices, websites) for but are not yet able to do, or problems that you would like to solve with technology?

*(Open)*

*Smart toilet*

*OnePlanet Research Center from Wageningen is developing a special toilet that will allow you to measure and monitor all sorts of things in the future. The toilet can take these measurements all by itself, you just have to sit on it, like a normal toilet. There are small measuring instruments in the toilet seat and in other places that can determine, for example, your heart rate, breathing, body temperature or substances in your urine or faeces. So you can learn all about your health just by using the toilet. This can be done via an app on your mobile, or if you prefer, through a direct connection with your doctor.
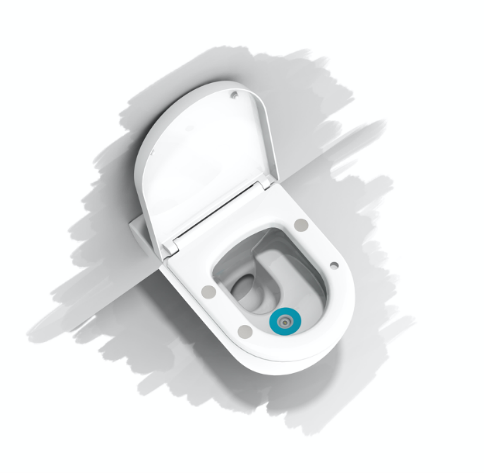
*

*The following questions are about what you think about this smart toilet.*

Question 24:

Would you like to use a smart toilet when dealing with your illness?

Yes

No

I do not know

Question 25:

Do you think a smart toilet could help you cope with your illness?

Yes -> question 26

No -> question 27

I don't know --> question 28

Question 26:

How do you think the smart toilet could help you? You can think of what you want to measure with the smart toilet, or which questions you could answer with the smart toilet. --> question 28

Question 27:

Why do you think the smart toilet couldn't help you?

Question 28:

When the smart toilet measures your toilet use, how would you prefer to receive it?

- I always want to be able to view my measurement data, for example in an app

- I would like to see a summary of relevant measurement data on a regular basis

- I just want to know when the toilet measures something I have to deal with

- I don't want to know anything myself, but I prefer that the data be forwarded directly to my doctor or specialist

- No, I wouldn't use the smart toilet

Question 29:

Who of you should have access to your data?

*(multiple answers possible)*

- yourself

- your partner or others who care for you

- your doctor or specialist

- scientists who research IBD

- the maker of the toilet

- the insurer

- others, namely:

- no one

Question 30:

What objections might you see to using the smart toilet?

*(Open)*

Question 31:

Would you like to say something about this questionnaire or the smart toilet seat?

*(Open)*

This is the end of this questionnaire.

Thank you very much for filling in.
