# Supplementary material for: Investigating Patient Perspectives on Using eHealth Technologies for the Self-Management of Inflammatory Bowel Disease: Mixed Methods Study
Source: J Med Internet Res. 2024 Sep 6;26:e53512. doi: 10.2196/53512 (PMC11415722; doi:10.2196/53512)
Supplement: Multimedia Appendix 2 [file jmir_v26i1e53512_app2.pdf]

Disease Characteristics

Symptoms

- Stomach ache (478 mentions)
- Increased toilet visits (477)
- Fatigue (451)
- Diarrhea (438)
- Blood or slime in stool (398)
- Pain in joints (256)
- Tenesmus (201)
- Limited appetite (188)
- Psychological symptoms (145)
- Dry or inflamed eyes (94)
- Fever (67)
- Fistula (58)
- High heartrate (55)
- Other symptoms (41)
- Obstipation (4)

Comorbidities

- Joint complaints (250 mentions)
- No comorbidities (144)
- Chronic skin conditions (107)
- Other comorbidities (106)
- Psychological disorders, depression (88)
- Dental and oral problems (85)
- Osteoperosis (81)
- Lung diseases (80)
- Rheumatism (76)
- Eye conditions (51)
- Blood conditions (49)
- Trombosis (21)
- Cardiovascular diseases (20)
- Pancreatitis (16)
- Spondyloartritis (16)
- Bechterew's disease (14)
- IBS (9)
- PSC (9)
- Migraine (9)
- Cancer (7)

Self-Management

Predictors

- Stomach ache (71 mentions)
- Fatigue (70)
- Changed stool, diarrhea (41)
- More frequent toilet visits (37)
- Stress (32)
- Blood or slime in stool (21)
- Feeling less well (18)
- Less appetite (16)
- Other predictors (15)
- Agitated bowel (14)
- Bloated feeling (12)
- Fever (10)
- Anxious feeling (9)
- Increased complaints (8)
- Being more emotional (8)
- Nausea (6)
- Increased joint complaints (6)

Perceived_causes

- Stress (158 mentions)
- Diet and trigger foods (105 mentions)
- Work and life pressure (45)
- Other causes (17)
- Medication (14)
- Side-effects other illness (10)
- Alcohol (9)
- Lack of sleep (9)
- Weather conditions (6)

Self-management Needs

- Support (105 mentions)
- Information (81)
- Resilience, optimism (64)
- Action planning (35)
- None (35)
- Leading a normal life (8)

Self-management Strategies

- Rest, sleep, relaxation (65 mentions)
- Healthy diet, avoid trigger foods (51)
- Stress avoidance (36)
- Adapt medication (28)
- Acceptance (23)
- Physical activity (21)
- Adapt lifestyle (17)
- Regularity (13)
- Professional help (5)
- Other (5)

ICT Use in Self-Management

Hardware use

- No solution fits my needs (119 mentions)
- Smartphone (116)
- Wearable tracker (108)
- Smartwatch (99)
- Computer / Tablet (26)
- Domotica / 'Smart' objects (11)

Software use

- MijnIBDcoach (99 mentions)
- IBD information websites (47)
- Apps provided by hospital (46)
- Generic health apps (32)
- Nutrition apps (24)
- Activity apps (20)
- Hogenood app (16)
- Diary apps (11)
- IBDream app (10)
- Facebook (8)
- Stooltracking apps (8)
- Scoial media, general (8)
- Relaxation apps (7)
- Sleep apps (5)
- DEARhealth app (4)
- Period tracker apps (3)
- Calprosmart app (2)
- Buikbuddy app (2)
- Medication tracker apps (2)
- Apps for other conditions (2)
- Pooptracker app (2)
- Cognition training apps (1)
- Mood tracker apps (1)
- Bowelle app (1)
- Podcasts (1)

Perceived use cases ICT

Theme: Learning about condition
- Documenting condition (153 mentions)
- Learning about condition (93)
- Diagnosing condition (24)

Theme: Living with condition
- Connecting with care professionals (107)
- Activity tracking / keeping active (88)
- Reducing burden medical care (41)
- Finding toilets (24)
- Directive tracking / behaviour change (23)
- Supporting healthy diet (19)
- Medical profession should do more (7)

Theme: Emotional and social support
- Connecting with peers (33)
- Staying calm (14)
- Detecting stress (5)

The Smart Toilet

Perceived use cases smart toilet

Theme: Learning about condition
- Documenting condition (178 mentions)
- Signalling changes (142)
- Diagnosing condition (103)
- Measuring inflammation markers (79)
- Measuring calprotectine (71)
- Measuring stool composition (54)
- Measuring urine composition (20)
- Learning about condition (8)

Theme: Living with condition
- Directive tracking / behaviour change (68)
- Reducing the burden of medical care (40)
- Tailoring treatment to my situation (5)
- Love of technology (2)

Theme: Negative use cases
- No need (93)
- I don't know (48)
- I would rather not use the toilet (35)
- I cannot use it because of my stoma (18)
- (I don"t want to use it because) Current advice suffices (12)

Data agency

- I want the data to be always available to me (418 mentions)
- I want reports only when action is needed (123)
- I want regular summaries of relevant data (58)
- I want reports when action is needed to go directly to my health care provider (23)

Perceived concerns

Theme: Privacy and data agency
- Privacy (142 mentions)
- Agency control (52)

Theme: Practical concerns
- Cost (85)
- Can others use the toilet as well? (70)
- Breaking down, tech issues (60)
- Too much hassle (52)
- Unpleasant (18)
- Hygiene (9)
- Mostly using other toilets (7)
- Aesthetics and design of toilet (3)

Theme: Concerns in dealing with the condition
- Unnecessary worries (76)
- Constant reminder of illness (51)
- Overmedicalisation of condition (32)
- Replacement of contact with health care by technology (18)

Theme: Validity
- Won't work (for me) (69)
- Reliability and validity of measurements (64)
- Unnecessary data generation (29)

Doubts and wishes

- I will not use the toilet (112 mentions)
- I would like to try it right away (54)
- I would like a bidet installed (17)
- I doubt I would use it (3)
